# Pilot feasibility study to deliver low-dose CT lung cancer screening in Scotland: descriptive findings from the LungScot study

**DOI:** 10.1136/bmjopen-2026-120569

**Published:** 2026-09-15

**Authors:** Debbie Cavers, Ahsan R Akram, Elizabeth K Sage, John D Maclay, Gordon W Cowell, James Mitchell, Graeme Dickie, Christine Campbell, Kathryn A Robb, Frank Sullivan, Peter Murchie, Aileen Neilson, Robert J C Steele, Edwin J R van Beek, David Weller

**Affiliations:** 1 Usher Institute, University of Edinburgh, Edinburgh, UK; 2 Centre for Inflammation Research, The University of Edinburgh The Queen’s Medical Research Institute, Edinburgh, UK; 3 Respiratory Medicine, NHS Highland, Inverness, UK; 4 Glasgow Royal Infirmary, NHS Greater Glasgow and Clyde, Glasgow, UK; 5 School of Health and Wellbeing, University of Glasgow, Glasgow, UK; 6 Respiratory Medicine, Aberdeen Royal Infirmary, NHS Grampian, Aberdeen, UK; 7 Beatson West of Scotland Cancer Centre, Glasgow, UK; 8 University of St. Andrews, St Andrews, UK; 9 Department of Family and Community Medicine & Dalla Lana School of Public Health, University of Toronto, Toronto, Ontario, Canada; 10 University of Dundee, Dundee, UK; 11 University of Galway, Galway, Ireland; 12 Division of Applied Health Science, University of Aberdeen, Aberdeen, UK; 13 Edinburgh Clinical Trials Unit (ECTU), Usher Institute, The University of Edinburgh, Edinburgh, UK; 14 Institute for Neuroscience and Cardiovascular Research, University of Edinburgh, Edinburgh, UK

**Keywords:** Lung Neoplasms, Mass Screening, RADIOLOGY & IMAGING

## Abstract

**Abstract:**

**Objectives:**

To assess the feasibility and acceptability of delivering risk-stratified low-dose CT (LDCT) lung cancer screening across diverse geographical and socioeconomic settings in Scotland and to characterise uptake, participant profile and screening outcomes.

**Design:**

Prospective, multicentre pilot feasibility study with descriptive analysis.

**Setting:**

Primary and secondary care interface across four regional Health Boards in Scotland, encompassing urban, rural, remote and socioeconomically deprived populations.

**Participants:**

2801 individuals aged 50–74 years, identified from primary care electronic medical records (EMRs), with a history of smoking were invited (current or within 15 years where available); 668 (23.8%) responded. Of 608 assessed for eligibility (50.8% female; median age 63 years), 503 (82.7%) met at least one predefined high-risk criterion.

**Interventions:**

Invitation to a lung health check, followed by telephone-based risk assessment using US Preventative Services Task Force, Liverpool Lung Pathway version 2 and the Prostate, Lung, Colorectal and Ovarian Screening Trial criteria. High-risk participants were offered a one-off LDCT scan at local centres.

**Primary and secondary outcome measures:**

Primary: feasibility indicators including uptake, risk eligibility and scan completion. Secondary: prevalence of lung cancer, pulmonary nodules and incidental findings (eg, coronary artery disease, emphysema).

**Results:**

Response rate was 23.8% (668/2801), varying from 17.8% to 38.1% across practices and lower in more deprived areas. Of 503 eligible participants, 436 (86.7%) underwent LDCT. Abnormal findings were present in 83.3% of scans. Lung nodules were detected in 25.7% (112/436), with 11.8% requiring follow-up. Lung cancer was diagnosed in 1.15% (5/436; 95%), alongside five additional malignancies. Coronary artery disease was identified in 56.4% (246/436) and emphysema in 33% (144/436). Non-responders were more likely to be current smokers (69.3% vs 49.8%).

**Conclusions:**

Recruitment of people for lung cancer screening using the smoking history recorded in their primary care EMR is feasible. This approach identifies a high-risk population with a high burden of both malignant and non-malignant disease, and with substantial undiagnosed disease burden. However, uptake was modest and socially patterned, highlighting the need for equity-focused implementation strategies, including repeated invitations and targeted engagement. These findings are particularly relevant as Scotland considers implementation of a national lung cancer screening programme within a health system characterised by marked geographical and socioeconomic inequalities. Further research should evaluate long-term outcomes, cost-effectiveness and strategies to improve equitable participation.

**STRENGTHS AND LIMITATIONS OF THIS STUDY**

- Low-dose CT lung screening covering diverse Scottish settings, including rural and socioeconomically deprived populations.
- Use of multiple validated risk prediction models aligned with established UK screening protocols.
- Strong patient and public involvement informing methodological study design and delivery.
- Modest uptake influenced by single invitation strategy without reminder systems or community outreach.
- Incomplete follow-up and inability to assess long-term outcomes or system-level impact.

## Background/introduction

 There is compelling global evidence that risk-stratified lung screening using low-dose CT (LDCT) scanning can reduce lung cancer mortality by upwards of 20%, through identifying cancers at an earlier stage when treatment options and outcomes are more favourable.^1 2^ A number of trials conducted in the USA and Europe dating back over 20 years have contributed to this evidence base. In the USA, the National Lung Screening Trial provided early evidence,^3^ while in Europe, the Nederlands-Leuvens Longkanker Screenings ONderzoek (NELSON) trial is one of the largest of its kind,^4^ and lung screening trial activity in England includes the UK Lung Screening Trial,^2^ the Yorkshire Lung Screening Trial^5^ and the SUMMIT study.^6^ As a result, lung screening of at-risk groups is slowly being introduced globally.^7 8^ In the UK, the National Screening Committee has recommended that lung screening be implemented, with currently variable delivery across the devolved nations. National Health Service (NHS) England commenced the roll-out of the Targeted Lung Health Check (TLHC) programme in April 2019. As of March 2025, 7193 lung cancers had been detected through the TLHC programme since its launch in 2019, with 76% diagnosed at the earliest stages (stages 1 or 2) when treatment is potentially curative.^9^ The programme is being rolled out nationwide by 2030,^10^ and expansion is prioritising high-deprivation areas in order to reduce inequalities.^11^ The programme is now called the NHS Lung Cancer Screening Programme reflecting its broader rollout and integration into national screening policy.^12^ At present there is no similar activity in Scotland or Northern Ireland, although a programme is due to be implemented in Wales in 2027.^13^ The current lack of a coordinated programme for Scotland raises questions of feasibility, equitable access and system readiness with Scotland’s unique geography and NHS organisation.

While the evidence for the effectiveness of lung screening in reducing lung cancer mortality is widely accepted, health systems face challenges in identifying optimal strategies for implementation of lung screening. There are multiple dimensions to consider, including accessibility, IT and infrastructure and workforce and capacity issues.^8 14–16^ Real-world mortality benefit is contingent on successful implementation and uptake across diverse groups. Scotland’s combination of remote and rural populations, marked socioeconomic inequalities and challenges within primary care infrastructure presents unique implementation challenges that may not be directly comparable to existing pilots focused in urban centres. Lung screening uptake varies in different studies and target populations—addressing this variation by understanding how lung screening might operate in Scotland’s diverse geography, population distribution and health service organisation, is also important in lung screening implementation.^17–20^

The LungScot study tested the feasibility and acceptability of risk stratified lung screening using LDCT in Scotland. We aimed to adapt the established lung health check model to the distinctive Scottish context, where patterns of rurality, deprivation and health service organisation differ from elsewhere in the UK, addressing an important gap in the UK evidence thus far. In particular, understanding how participant identification, engagement and screening delivery would operate in Scotland’s mixed urban–rural Health Boards is critical for equitable implementation. Our objectives were to examine the process of identifying eligible participants from primary care records, to map and evaluate the screening pathway and to test delivery in areas reflecting Scotland’s particular challenges of geography and inequality. In doing so, we can inform further research, policy and service delivery in this area. This paper reports descriptive findings from the pilot feasibility implementation of LDCT lung screening across four Scottish Health Boards, providing evidence to inform future implementation of a national Scottish lung screening programme.

## Methods

### Participants, sampling and recruitment

**Figure 1:**
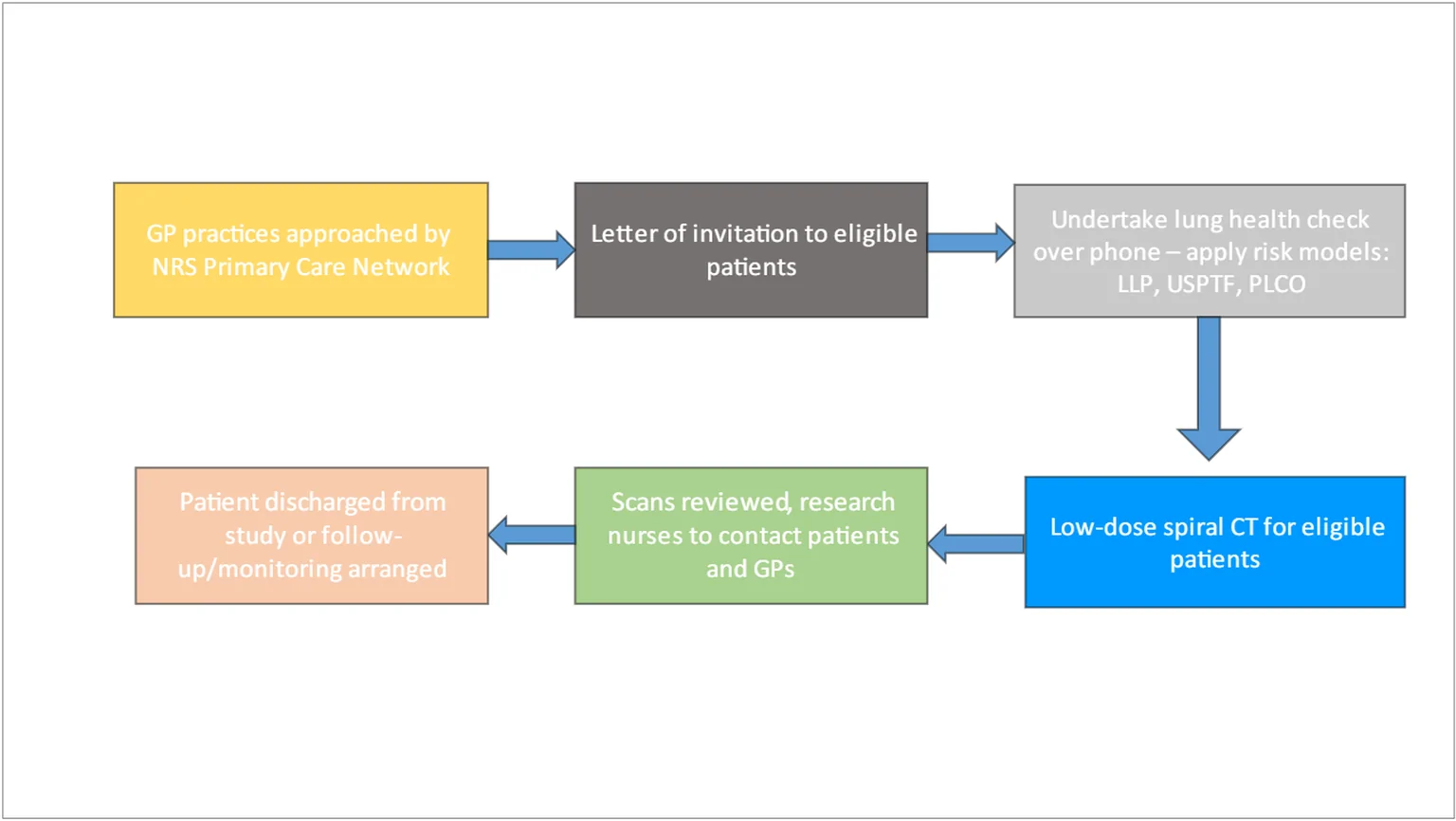
Lung health check participant pathway. GP, general practitioner; LLP, Liverpool Lung Pathway version 2; NRS, National Research Scotland; PLCO, Prostate, Lung, Colorectal and Ovarian; USPTF, US Preventative Services Task Force.

Eligible participants were identified and a sample selected from the primary care electronic medical records (EMRs) of participating practices, assisted by the National Research Scotland Primary Care Network co-ordinators.^21^ Selection was based on age (50–74 years) and smoking status (ever smoker, with smoking within the past 15 years where this information was available in primary care records). A proportion of eligible participants were selected from each practice list; numbers were capped in each practice (ranging from 200 to 350 patients) and sample size was guided by anticipated response rate, limited resources to provide scans and the inclusion of participants across a range of practice sizes, locations and deprivation indices. Participating general practitioner (GP) practices were recruited from NHS Lothian, NHS Highland, NHS Greater Glasgow and Clyde (GGC) and NHS Grampian, chosen to cover areas with a wide range of sociodemographic characteristics including urban, rural, remote and deprived areas. NHS Lothian served as the lead site where the first phase of recruitment took place, and the study was subsequently expanded to the additional three regional Health Boards to widen the inclusion of deprived and rural areas. The participant pathway is shown in figure 1. Potential participants were sent a postal letter of invitation offering them a ‘lung health check’, including an information sheet and paper response form with FREEPOST return envelope to indicate their interest in taking part. The researcher (DC) or a research nurse made contact with respondents by telephone to discuss the study, agree verbal consent (consent forms were subsequently sent to participants to sign and return), and assess their risk-based eligibility to take part, according to the criteria of three validated risk tools (US Preventative Services Task Force (USPSTF), Liverpool Lung Pathway version 2 (LLPv2) and the Prostate, Lung, Colorectal and Ovarian Screening Trial criteria (PLCOm2012)). Those meeting any of the risk tool threshold and other eligibility criteria were offered a one-off LDCT scan at their nearest participating hospital. Local research nurses coordinated scan appointments. Scans were interpreted and reported by study thoracic radiologists. Results were forwarded to respiratory clinicians who, in collaboration with research nurses, communicated results to participants by letter, with copies sent to their GPs, who coordinated any follow-up within primary care. The clinical study teams coordinated cases where any follow-up was required in secondary care, including follow-up scans, nodule management and urgent suspicion of cancer, by referring them into NHS pathways for care, following British Thoracic Society guidelines for the management of pulmonary nodules where appropriate.^22^ Participants were assigned a study ID (linked to their Community Health Index (CHI) number), to track their progress through the study. Descriptive data on age, sex, smoking status, Scottish Index of Multiple Deprivation (SIMD) and number of chronic conditions were collected on respondents and non-respondents where possible. Smoking cessation advice, signposting participants to the Scottish ‘Quit Your Way’ initiative^23^ was offered to patients on exit from the study in NHS Lothian only.

### Risk assessment

Eligibility for LDCT was determined using predefined risk thresholds aligned with the NHS England THLC protocol, ensuring consistency with established UK practice.^24^ Three validated tools were used to assess participants’ risk of developing lung cancer. Two of these were risk prediction models: the LLPv2 and the PLCOm2012 (16, 17). Participants were classified as high-risk using model-specific thresholds: a 5-year predicted risk of ≥2.5% for the LLPv2 and a 6-year predicted risk of ≥1.51% for the PLCOm2012. A version of the PLCOm2012 adapted for UK populations was applied to account for local ethnic group distributions. Permission to use both models was obtained for this study. The information included in the two models is detailed in table 1.

**Table 1:** Factors included in two multivariable risk prediction models^24^

LLPv2: ≥2.5% risk	PLCO_M2012_: ≥1.51% risk
Age Gender Smoking duration (years) Previous pneumonia/COPD/emphysema/bronchitis/TB Occupational asbestos exposure Previous history of malignancy Previous family history of lung cancer and relative’s age at onset, that is, <60 years or >60 years; whether first-degree relative	Age (years) Education level Body mass index COPD/chronic bronchitis/emphysema Personal history of lung cancer Ethnicity Smoking status Average number of cigarettes smoked per day Duration smoked (years) Years having ceased smoking

COPD, coronary obstructive pulmonary disease; LLPv2, Liverpool Lung Pathway version 2; PLCOm2012, Prostate, Lung, Colorectal and Ovarian Screening Trial criteria; TB, Tuberculosis.

The USPSTF criteria from 2021 was also used, whereby participants were considered ‘high-risk’ if they fell within the age bracket 50–80 years and reported that they were current smokers or had smoked within the last 15 years, with a history of more than 20 pack years**.^25^

Participants were considered eligible for a lung health check if they met the threshold for any one of the three risk tools to ensure the lowest threshold for inclusion.

### The Lung Health Check

The Lung Health Check was a two-stage process, closely modelled on the Lung Health Checks delivered in NHS England, according to the Standard Protocol for the Lung Health Check Programme.^24^ In the first stage, risk-based eligibility was established over the phone incorporating the three risk prediction tool algorithms, and findings were recorded on individual data capture forms. Using the risk information generated by the algorithm, the researcher informed participants over the phone whether or not they met the criterion to be offered a LDCT scan. If the offer was accepted, participants’ details (including the data capture form and signed consent form) were uploaded to an NHS research platform (NHS Edge) to securely link with their NHS health record and participants were informed that a member of the team would be in touch to arrange a CT scan appointment (by phone or letter, variable by health board) at their nearest participating imaging centre. Participants were given the opportunity to ask questions, and a further information sheet with more details of the scan procedure was sent to participants with their confirmed appointment. Scan appointments were facilitated by attending radiographers and lasted approximately 15–20 min. Scan results were stored on patients’ electronic health records and recorded on data capture forms for the study. Follow-up for any incidental findings requiring secondary care action was within NHS settings and pathways.

### Patient and public involvement

An individual with a personal diagnosis of lung cancer (GD) is a co-investigator on the study and co-author on this paper, having been involved in the study from its inception. Patients and carers with experience of lung and other cancers were convened as a patient advisory group to consult on all aspects of the study.

### Data and analysis

Participants’ sociodemographic characteristics (sex, age, SIMD, self-reported smoking status, number of health conditions), risk scores and scan results were recorded on a purpose-designed data capture form. Information was collated and subject to descriptive analysis, reported as counts and percentages. Limited sociodemographic information was collected on non-responders by the NRS PCN from primary care records (gender, age, SIMD, last recorded smoking status where available) and descriptively summarised. Scans were reported by thoracic radiologists in each health board area with more than 15 years’ experience in a structured format and incidences of abnormalities recorded.

## Results

Descriptive findings from the intervention are reported below.

### Uptake, participant characteristics and risk scoring

**Figure 2:**
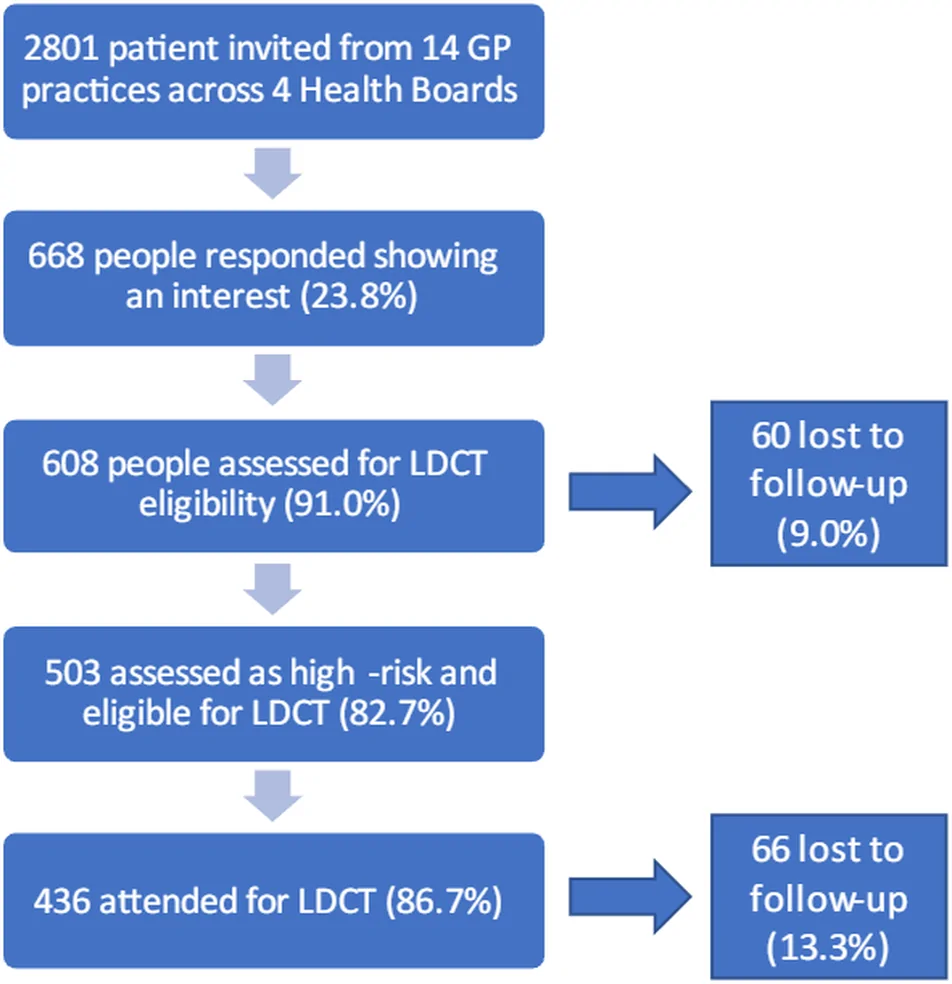
Participant response at each stage of the intervention. GP, general practitioner; LDCT, low-dose CT.

A total of 2801 patients were invited between May 2022 and October 2024 to take part in the study across the four Health Boards, with 668 patients responding to the invitation letter to express interest in the study (23.8%). Figure 2 shows participant response at each stage of the intervention. Response rates varied across the 14 participating practices from 17.8% to 38.1%. Lower response rates were observed in practices located in areas of high socioeconomic deprivation (table 2); for example, response rates were 35.1% in one of the least deprived quintiles in NHS Highlands, and 17.8% in one of the most deprived in NHS Grampian. There was a greater prevalence of existing conditions and multimorbidity among participants in NHS Grampian compared with NHS Lothian and NHS Highlands where overall response rates were also lower (no data are available from NHS GGC). Table 2 reports a breakdown of response rates by SIMD, and the number of existing health conditions in the four Health Boards (see online supplemental table 1 for breakdown by practice).

This socioeconomic gradient in participation was evident across the participating Health Boards and represents an important consideration for future programme implementation.

Of the responders, data were obtained from 608 participants (309 females (50.8%) and 299 males (49.2%), a median age 63 years), while 60 did not engage with follow-up to assess eligibility for a scan. Three hundred and five participants (50.2%) were past smokers and 303 were current smokers (49.8%); 85% of participants had been smoking for more than 30 years (see online supplemental table 2) for self-reported smoking by health board region). While smoking was more prevalent in NHS Grampian where socioeconomic deprivation is high among participants, this was not observed in NHS GGC practices where there are similar levels of deprivation.

**Table 2:** Recruitment response to initial invitation and SIMD in four Scottish Health Boards

Health board	Response rate	Average age (years)	Sex (male/female)	Current smokers/ever smokers	SIMD quintiles (1–5) n %	No. of existing conditions (0–5+) n %
Lothian	1528 invited, 379 responses (24.8%)	63	171 males (50.35) 169 females (49.7%)	155 current (45.6%) 185 ever (54.4%)	27, 7.3% 2–109, 29.5% 3–103, 27.8% 4–84, 21.9% 5–45, 13.2 (n=11 missing data)	169, 44.6% 1–115, 30.4% 2–60, 15.8% 3–26, 6.9% 4–8, 2.1% 5+ −1, 0.3%
GGC	418 invited, 77 responses (18.35%)	62	30 males (42.9%) 40 females (57.1%)	32 current (45.7%) 38 ever (54.3%)	53, 68.4% 2–16, 22.0% 3–3, 3.9% 4–2, 2.6% 5–2, 2.6% (n=1 missing data)	Data not available (n=77 missing data)
Grampian	313 invited, 59 responses (18.9%)	63	21 males (36.8%) 36 females (63.2%)	38 current (66.7%) 19 ever (33.3%)	18, 30.5% 2–12, 20.3% 3–21, 35.6% 4–8, 13.6% 5–0, 0%	17, 28.8% 1–22, 37.3% 2–10, 16.9% 3–6, 10.2% 4–2, 3.4% 5+ - 2, 3.4%
Highlands	542 invited, 153 responses (30.1%)	62	77 males (54.6%) 64 females (45.4%)	78 current (55.3%) 63 ever (44.7%)	14, 9.2% 2–45, 29.4% 3–51, 33.3% 4–43, 28.1% 5–0, 0%	82, 53.4% 1–39, 25.5% 2–21, 13.7% 3–7, 4.6% 4–3, 2.0% 5+ - 1, 0.7%
Totals	2801 invited, 668 responses (23.8%)	63	299 males (49.2%) 309 females (50.8%)	303 current (49.8%) 305 ever (50.2%)	1-112, 17.1% 2-182, 27.8% 3-178, 27.2% 4-136, 20.8% 5-47, 7.2%	268, 45.3% 176, 29.8% 91, 15.4% 39, 6.6% 13, 2.2% 5+ - 4, 0.7%

GGC, Greater Glasgow and Clyde; SIMD, Scottish Index of Multiple Deprivation.

**Figure 3:**
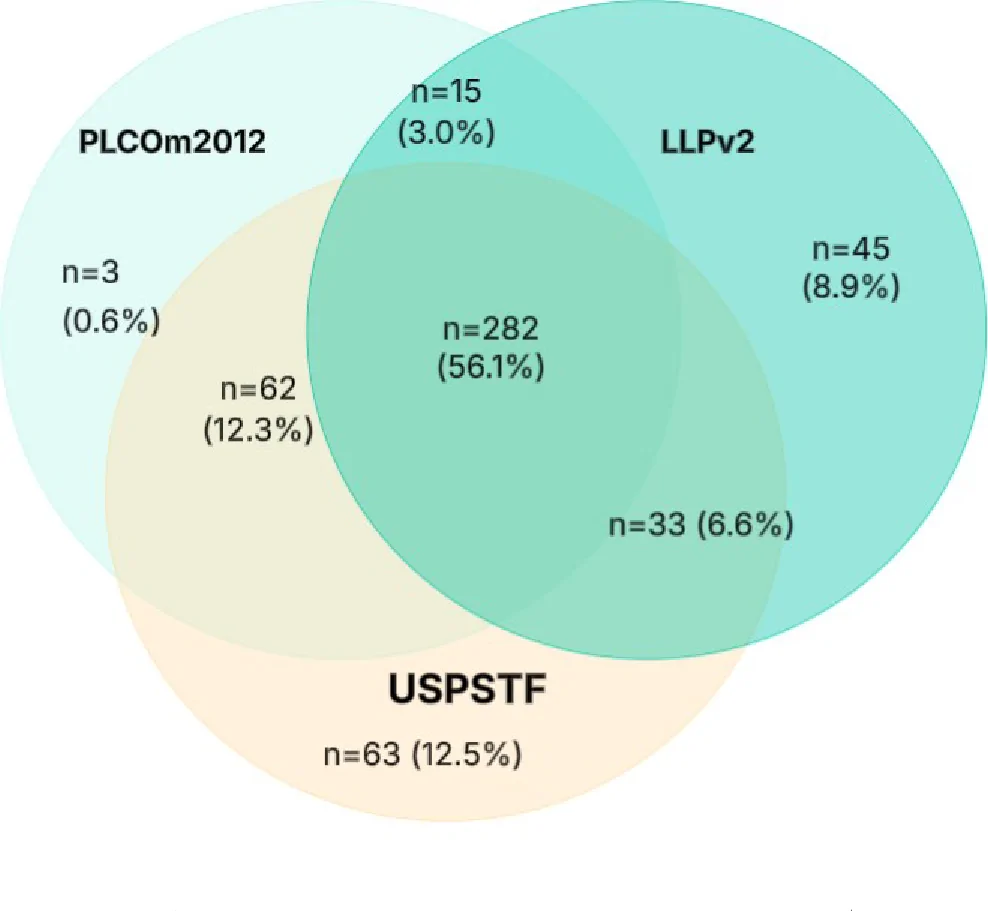
High-risk participants meeting criteria for each risk assessment tool (n=503). LLPv2, Liverpool Lung Pathway version 2; PLCOm2012, Prostate, Lung, Colorectal and Ovarian Screening Trial criteria; USPSTF, US Preventative Services Task Force.

When applying study inclusion criteria, the USPSTF criteria was the most commonly met threshold, followed by the LLPv2 and PLCOm2012 criteria (table 3). Figure 3 shows the number of participants to meet criteria for each of the risk assessment tools (online supplemental table 3 shows this data in table form). From the risk assessment, 115 (18.9%) self-reported a known diagnosis of coronary obstructive pulmonary disease, 23 (3.8%) reported having emphysema, 38 (6.3%) cancer and 77 participants (12.7%) reported asbestos exposure. Overall, 503 participants (82.7%) met the criteria for one or more of the risk assessment tools and were classified as high-risk, and therefore eligible for an LDCT scan.

**Table 3:** Risk criteria assessment of eligible individuals (n=608)

	LLPv2	PLCOm2012	USPSTF
Criteria met	375 (61.7%)	362 (59.5%)	440 (72.4%)
Criteria not met	233 (38.3%)	246 (40.5%)	168 (27.6%)

LLPv2, Liverpool Lung Pathway version 2; PLCOm2012, Prostate, Lung, Colorectal and Ovarian Screening Trial criteria; USPSTF, US Preventative Services Task Force.

### Scan findings

LDCT was successfully performed in 436/503 (86.7%) participants, with 67/503 (13.3%) participants not attending for a planned CT study, including those not attending on more than one occasion. Of those scanned, only 73 (16.7%) were classed as ‘normal’ scans according to the reporting criteria. Two lung cancers were diagnosed at baseline scanning, with a further three identified following subsequent follow-up scans (overall incidence 1.15%; baseline prevalence 0.46%). The two baseline lung cancers were both stage IIB, and the remaining lung cancers were stage IA, IIIC and IVB. The LDCT also detected five other cancer types (two breast, 1 liver, and neuroendocrine cancer). Nodules were detected in 112 cases (25.7%). Of these, 61 (54.5%) were classed as non-significant or not requiring follow-up in accordance with national guidance,^22^ and 49 (43.8%) required follow-up scans (n=2 were unclassified). Table 4 shows the breakdown of non-significant and actionable nodules by health board region. NHS Grampian and NHS GGC observed the highest proportions of actionable nodules compared with NHS Lothian and NHS Highlands.

**Table 4:** Breakdown of non-significant versus actionable nodules by health board region

Health board	Lothian	GGC	Grampian	Highlands	Totals
Nodule type					
No nodule	165 (77.1%)	29 (82.6%)	24 (46.1%)	87 (76.3%)	305 (73.5%)
Non-significant nodule	29 (13.6%)	2 (5.7%)	14 (26.9%)	16 (14.0%)	61 (14.7%)
Actionable nodule	20 (9.3%)	4 (11.4%)	14 (26.9%)	11 (9.6%)	49 (11.8%)
Totals	214	35	52	114	415

GGC, Greater Glasgow and Clyde.

#### Incidental findings

The most commonly detected incidental finding from participant LDCT scans was coronary artery disease (CAD). CAD was detected in 246 cases (56.4%). The majority of CAD was classified as mild (n=139; 56.5%), 82 cases (33.3%) were classed as moderate, 25 (10.2%) were moderate/severe or severe and 5 (2%) were unclassified. Table 5 reports the breakdown of CAD in the different Health Boards. Table 6 shows the breakdown of emphysema by health board region. Other incidental findings of note included granulomas, scarring (including linear, postoperative and postinfective scarring), pleural plaques, thoracic aneurysm, hiatus hernia, calcified aortic valve, gallbladder stones and pulmonary fibrosis.

**Table 5:** Coronary artery disease (CAD) by health board region

	Mild	Moderate	Mod/severe or severe	Totals	Total scans	% CAD
Health board						
Lothian	63, 58.9%	36, 33.6%	8, 7.5%	107	215	49.8
GGC	16, 61.5%	6, 23.1%	4, 15.4%	26	55	47.3
Grampian	21, 67.7%	7, 22.6%	3, 9.7%	31	52	59.6
Highlands	39, 47.5%	33, 40.2%	10, 12.2%	82	114	71.9
Totals	139	82	25	246	436	56.4%

GGC, Greater Glasgow and Clyde.

**Table 6:** Emphysema by health board region

Health board	Emphysema	Percentage
Lothian	41	19.1
GGC	21	38.2
Grampian	28	53.8
Highlands	54	47.4
Totals	144	33% of all scans

GGC, Greater Glasgow and Clyde.

### Non-responders

Overall, the demographic characteristics of the non-responder groups (ie, those who did not respond to the invitation to a lung health check) across the Health Boards were similar to those who agreed to participate, although no statistical comparisons were made. Overall, responders and non-responders were similar in terms of age, sex and SIMD (where data are available; see online supplemental tables 4–7 for more detail). There were more current smokers among non-responders than past smokers recorded in the primary care records (69.3% of non-responders recorded as current smokers compared with 49.8% current smokers among responders). There was an even spread across deprivation quintiles 1–3 across all Health Boards combined, but non-responders in NHS GGC and NHS Grampian had higher rates of deprivation (falling in the lowest quintile) than Lothian and Highland counterparts. Similar patterns of deprivation can be seen among non-responders as compared with responders to the lung health check (almost 90% of non-responders fell in the bottom two SIMD quintiles for NHS GGC and around 64% for NHS Grampian, compared with 41% and 38% for NHS Lothian and the NHS Highlands, respectively). Unfortunately, these data are not available for NHS GGC non-responders, where rates of deprivation are highest. Interpretation of non-responder characteristics is therefore limited by incomplete data availability across Health Boards and the absence of formal statistical comparison.

## Discussion

### Summary of findings

This study represents the first Scottish pilot feasibility study of risk-stratified lung cancer screening using LDCT, delivered across four different Health Boards. We demonstrate that it is feasible to identify, assess, and scan a population at higher levels of risk within existing NHS Scotland structures, including people in geographically dispersed and socioeconomically diverse settings.^26^ Although overall response to the initial invitation was modest (23.8%), the majority of responders met validated risk criteria (82.7%) and a substantial burden of clinically relevant disease was detected, including lung cancer, pulmonary nodules, CAD and emphysema. As a feasibility study, these findings primarily inform the practicalities of delivery and implementation of the recruitment and investigation stages, rather than screening effectiveness or long-term clinical outcomes.

The observed lung cancer detection rate (1.15%) and nodule prevalence are consistent with those reported in early prevalence rounds of other UK and international screening programmes.^3 4^ Importantly, the high prevalence of smoking-related comorbidity reinforces the value of lung screening as a broader ‘lung health check’, rather than a cancer-only intervention. These comparisons should be interpreted in the context of differences in programme design, invitation strategy and repeat screening rounds across settings. At the same time, the strong socioeconomic patterning of uptake highlights the central implementation challenge facing any future Scottish lung screening programme: how to ensure that implementation does not exacerbate existing health inequalities.

### Comparison with existing evidence

Response rate and scan uptake in this pilot feasibility study was lower than that now achieved by England’s TLHC programme, where attendance exceeds 50% in many areas.^27^ However, this difference is unsurprising. Early TLHC pilots reported uptake rates comparable to ours when invitations were single, non-repeated and unsupported by large-scale public messaging or community outreach.^5 28^ Moreover, the LungScot pilot invited people to a research study as opposed to an NHS screening programme. Evidence from England and beyond demonstrates that uptake improves substantially with iterative invitation rounds, endorsement from primary care and locally tailored engagement strategies—particularly when screening is framed around ‘lung health’ rather than cancer alone.^14 15 29^

The strong association between deprivation and lower participation observed here mirrors findings across the UK, Europe and North America. Individuals living in more deprived areas, current smokers and those with multimorbidity are consistently less likely to engage with screening despite being at highest risk of lung cancer mortality.^18 19 30^ These findings reinforce international consensus that equity-focused design is not optional but fundamental to effective lung screening implementation and is consistent with Scottish screening policy.^8 31 32^ Design features likely to influence equitable uptake include the invitation approach, location of scanning facilities, flexibility of appointments and primary care endorsement.

Our use of multiple validated risk tools (USPSTF, LLPv2, PLCOm2012) aligns with international best practice and reflects ongoing debate regarding optimal eligibility criteria. The high overlap observed between tools is consistent with findings from English and European studies and supports the pragmatic use of combined or simplified risk approaches in real-world programmes.^2 16^

Incidental findings—particularly CAD and emphysema—were common, echoing reports from NELSON, UKLS and TLHC cohorts. This international experience highlights the importance of recognising the need to balance preventive potential against incidental findings generating additional workload and patient anxiety, concerns highlighted in corresponding qualitative work (manuscript in preparation). There is growing recognition that LDCT screening offers a secondary prevention opportunity for cardiovascular disease, smoking-related lung disease and multimorbidity more broadly.^8 14^ However, international experience also cautions that incidental findings increase downstream workload and must be managed through clear protocols to avoid unnecessary investigation and anxiety.

A major strength of this study is its role as Scotland’s first practical test of LDCT lung screening delivery. Unlike many earlier pilots, LungScot was explicitly designed to reflect Scotland’s unique context, incorporating urban, rural and remote populations and areas with high socioeconomic deprivation. Demonstrating feasibility across these settings is a critical prerequisite for any future national programme.

Additional strengths include the use of multiple validated risk models, close alignment with established TLHC protocols and integration with routine NHS pathways for follow-up and management. Patient and public involvement from study inception further strengthens the relevance and acceptability of the work.

There are, however, important limitations. Uptake was modest, reflecting participants being invited to a research study rather than an NHS service, limited study resources, the absence of repeat and reminder invitations, call–recall systems, mobile scanning units and sustained public engagement—features now recognised as essential for equitable delivery. Attrition at different stages of the pathway limited complete follow-up for some participants, including those identified as high-risk group who did not attend their scan appointment. We were also unable to determine whether conditions such as emphysema or CAD were previously diagnosed, limiting interpretation of the ‘new’ disease burden identified. Radiological assessments of the severity of CAD and emphysema are subjective and may vary between radiologists, which could influence categorisation of these findings and adds weight to the need to incorporate robust, quantitative radiology reporting measures. We are also unable to capture longer-term downstream impact on primary care nor ethnicity data on participants. Finally, as a feasibility study, LungScot was not designed to assess stage shift, cost-effectiveness or mortality impact.

### Implications for future implementation in Scotland

Scotland’s later adoption of lung screening—while often framed as a disadvantage—arguably presents a unique strategic opportunity to design a programme that is implementation-ready from the outset. Unlike early-adopting regions, Scotland can draw directly on over a decade of international evidence and real-world learning from England’s TLHC programme, enabling known pitfalls to be avoided and best practice to be embedded from the outset.^8 15^ This pilot provides an initial evidence base from within the Scottish NHS on which those implementation decisions can now build. Further evidence from improved EMR algorithms, repeated screening rounds, cost-effectiveness analyses and workforce modelling would be required to inform decisions about national implementation.^33 34^ In future risk prediction models, biomarkers and artificial intelligence are likely to produce additional gains.^35^ In addition, Scotland’s existing national infrastructure—including the Scottish National Picture Archiving Communication Service and the Scottish National Radiology Reporting Service—could streamline radiological acquisition, reporting and quality assurance at scale.

A future Scottish programme will need to be explicitly designed around the characteristics and needs of the population it serves rather than assuming transferability of models developed elsewhere, in particular a need to recognise the challenges offered by Scotland’s more mixed rural-urban geography. This includes addressing Scotland’s dispersed rural geography, where travel distance and access barriers may necessitate mobile or community-based scanning models; high levels of deprivation and smoking prevalence in many communities; and a strong policy consensus that new national initiatives must not widen health inequalities. Understanding the demographics of the proportion of non-attenders to scan appointments (13.3% of those who took up the offer of a scan—a higher proportion than the first screening round of the NHS TLHC programme)^27^ and their reasons for non-attendance, will also be important. Evidence increasingly suggests that targeted outreach, community partnerships, flexible appointment systems and trusted local endorsement are critical to achieving equitable uptake.^17 19 20 36^

Our findings also reinforce the importance of considering lung screening as part of an integrated prevention strategy. The high prevalence of CAD and emphysema suggests potential value in linking lung screening with smoking cessation support, cardiovascular risk assessment and long-term condition management—provided sufficient capacity, funding and governance structures are in place to avoid unintended system burden. Early investment in workforce planning, community engagement and evaluation infrastructure will be critical to ensuring that any future Scottish programme delivers both effectiveness and equity. Policy implications are summarised in box 1.

Box 1 **Key implications for Scottish policymakers**

- Design for equity from the outset: Any future Scottish lung screening programme must prioritise equitable uptake through targeted outreach, flexible delivery models (including mobile/community-based scanning) and trusted local endorsement to avoid widening existing socioeconomic inequalities.
- Use Scotland’s national infrastructure advantage: Existing national digital and radiology systems provide a strong foundation for high-quality, standardised delivery at scale but early investment in workforce capacity and governance will be essential.
- Embed screening within prevention pathways: The high burden of comorbidity identified supports integrating lung screening with smoking cessation, cardiovascular risk management and long-term condition care to maximise population health benefit without overburdening services.

The recommendations presented extend beyond the immediate empirical findings of this pilot. They reflect interpretation of the broader literature on lung cancer screening implementation, applied to the prevailing organisational, demographic and infrastructural landscape in Scotland.

## Conclusions

This pilot feasibility study provides the first Scottish evidence that risk-stratified LDCT lung cancer screening can be delivered using primary care smoking records and identifies a population with a substantial burden of previously undiagnosed malignant and non-malignant disease. While uptake was socially patterned and modest, the study provides critical real-world insights into how screening might be delivered equitably across Scotland’s diverse settings to reach uptake rates in more established UK settings.^9^

As Scotland now considers national implementation, delayed adoption should not be viewed solely as a disadvantage. Instead, it offers an opportunity to implement and evaluate a programme informed by robust international evidence, explicitly designed to address deprivation, rurality and inequality from the outset.^37^ Failure to act risks widening existing disparities in lung cancer outcomes between Scotland and the rest of the UK and Europe; thoughtful, equity-focused implementation offers the potential to do the opposite.

## Supplementary material

10.1136/bmjopen-2026-120569 online supplemental table 1

10.1136/bmjopen-2026-120569 online supplemental table 2

10.1136/bmjopen-2026-120569 online supplemental table 3

10.1136/bmjopen-2026-120569 online supplemental table 4

## Data Availability

Data are available on reasonable request.
