# Supplementary material for: Pilot feasibility study to deliver low-dose CT lung cancer screening in Scotland: descriptive findings from the LungScot study
Source: BMJ Open. 2026 Sep 15;16(9):e120569. doi: 10.1136/bmjopen-2026-120569 (PMC13583738; doi:10.1136/bmjopen-2026-120569)
Supplement: Supplementary file 1 [file bmjopen-2026-120569.reviewer_comments.pdf]

PEER REVIEW HISTORY

BMJ Open publishes all reviews undertaken for accepted manuscripts. Reviewers are asked to complete a checklist review form and are provided with free text boxes to elaborate on their assessment. These free text comments are reproduced below.

ARTICLE DETAILS

Title (Provisional)

Pilot feasibility study to deliver low dose CT lung cancer screening in Scotland: descriptive findings from the LungScot study

Authors

Cavers, Debbie; Akram, Ahsan; Sage, Elizabeth; Maclay, John D; GW, Cowell; Mitchell, James; Dickie, Graeme; Campbell, Christine; Robb, Katie; Sullivan, Frank; Murchie, Peter; Neilson, Aileen; RJC, Steele; Van Beek, Edwin; Weller, David

---

VERSION 1 - REVIEW

**Reviewer**	**1**
**Name**	**Philip, Keir**
**Affiliation**	**National Heart and Lung Institute**
**Date**	**09-Jun-2026**
**COI**	**None**

Many thanks for the opportunity to review this paper. Its an important and well written paper. I only have a few minor comments for clarity: Title is not completely clear. Is this best described as a ‘pilot intervention’ or a pilot feasibility study?

Abstract is generally clear and well structured. Ensure all abbreviations written in full when first used, including USPSTF, LLPv2 and PLCOm2012

Intro clear

Methods – also needs abbreviations written in full when first used (not only later in the paper)

Is the plan for the main role out to use all three risk tools as was done here?

More information on how non-responders to invite were chased up again. What different modes of contact were used? E.g. phone, email, post etc

What happened to people identified as having emphysema or other non-malignant conditions in the study? I see that there is recommendation for consideration of this in the main programme, but what happened to study participants?

Similarly, was smoking cessation provided or at least VBA?

Throughout the manuscript, there needs to be a bit more clarity when using the term ‘pilot’ as to whether this is pilot intervention, pilot study, etc.

**Reviewer**	**2**
**Name**	**Danila, Edvardas**
**Affiliation**	**Vilnius University, Clinic of Chest Diseases, Immunology and Allergology**
**Date**	**12-Jun-2026**
**COI**	**None**

The aim of the study and the information presented in the manuscript are very clear. The primary objective of the study was to evaluate a specific lung cancer screening program.

I have no major comments on the manuscript. The results are described in detail.

I have a suggestion regarding the discussion section of the manuscript. Furthermore, I believe my advice could be valuable to the Scottish lung cancer screening program from a technical standpoint and help increase the response rate among invited individuals.

The cornerstone of any program’s success is high participation among invited individuals.

Results from other studies show that when people are invited by phone or during an in-person visit, participation rates are much higher.

https://doi.org/10.1016/j.ctarc.2025.100889

https://doi.org/10.3390/cancers17121956

**Reviewer**	**3**
**Name**	**de Bock, Geertruida H.**
**Affiliation**	**University Medical Center Groningen**
**Date**	**13-Jun-2026**
**COI**	**None**

Review

This study tested if risk based lung cancer screening using low dose CT could be delivered across different regions in Scotland. The programme worked ok for people who took part actually, showing high rates of lung cancer detection and also many previously unknown lung and heart conditions, but overall participation was low (about 24%), and especially in more deprived areas and in higher risk groups like current smokers uptake was poor.

I have some problems with the conclusion that the programme is feasible. It is mainly useful for the people who comes to take part, but not really for the whole population, because the highest risk people are less likely to take part. Maybe it should be more clearly explained what "feasible" means in this context. Is it only that the programme can be organised and run in practice?

My feeling is that this study shows LDCT screening can be delivered in practice and can find important disease in a high risk subset, but it does not yet show that it works well as a population screening programme in a fair and effective way. The main problem is not the scanning itself, it is more getting the people who are most at risk to actually join, and also how to deal with all the follow up from so many abnormal results.

Even for people who did join, it can be asked if this is always a good screening test. The yield is high for lung cancer and other lung findings, so targeting seems good. But there is also a very high number of "abnormal findings", which can mean overdiagnosis burden, more follow up tests, anxiety for patients, and higher healthcare costs. Also for some of these findings we are not really knowing what the best treatment is.

I would also like to ask the authors to maybe rethink the strengths and limitations section after the abstract.

Maybe I missed it, but what was done when the different risk tools did not agree? In table 2 many patients did not meet the criteria. What are the consequences of this? Was there less cancer in the group where criteria were not met?

What was the follow-up time? Or was this only the findings on the first round? It could be the case that the lung cancer detection rate will be higher, as the diagnostic testing is not finished in all patients yet.

VERSION 1 - AUTHOR RESPONSE

Reviewer: 1
Dr. Keir Philip, National Heart and Lung Institute
Comments to the Author:
Many thanks for the opportunity to review this paper. It's an important and well written paper. I only have a few minor comments for clarity: Title is not completely clear. Is this best described as a 'pilot intervention' or a pilot feasibility study?
Sincere thanks for your positive feedback and points to help improve the paper. We have added the word 'feasibility study' to the title and removed 'intervention' for greater clarity on the study design.

Abstract is generally clear and well structured. Ensure all abbreviations written in full when first used, including USPSTF, LLPv2 and PLCOm2012.
Thank you. This makes sense. We have expanded all abbreviations in the abstract now.

Intro clear
Methods – also needs abbreviations written in full when first used (not only later in the paper)
These revisions to the manuscript have now been made on page 4 of the methods section.

Is the plan for the main role out to use all three risk tools as was done here?
All three tools are currently being used in England – we have added a reference to the NHS England protocol - but decisions on which tools to be used in Scotland are yet to be finalised.

More information on how non-responders to invite were chased up again. What different modes of contact were used? E.g. phone, email, post etc.
Thank you for highlighting this important point. In response, we have clarified the mode of contact on page 4 of the methods section. Reminder letters were not sent to non-responders. We have now explicitly acknowledged this as a limitation of the study due to limited resources (page 13).

What happened to people identified as having emphysema or other non-malignant conditions in the study? I see that there is recommendation for consideration of this in the main programme, but what happened to study participants?
Thank you for raising this point for clarification. We have provided more detail on page 4 to clarify that GPs coordinated any follow-up within primary care. We also state here that the clinical team referred patients requiring any secondary care follow-up into NHS pathways, including for non-malignant conditions.

Similarly, was smoking cessation provided or at least VBA?
Information on smoking cessation is included on page 4/5, with reference Scotland's 'Quit Your Way' initiative. Participants were signposted to this resource but a full intervention was not delivered. We have also acknowledged this in the discussion and the importance of smoking cessation in a future programme.

Throughout the manuscript, there needs to be a bit more clarity when using the term 'pilot' as to whether this is pilot intervention, pilot study, etc.
Thank you for this feedback to help add consistency and clarity throughout the paper. We have now made revisions throughout the manuscript to achieve this.

Reviewer: 2
Dr. Edvardas Danila, Vilnius University, Vilnius University Hospital Santaros Klinikos
Comments to the Author:
The aim of the study and the information presented in the manuscript are very clear. The primary objective of the study was to evaluate a specific lung cancer screening program.
I have no major comments on the manuscript. The results are described in detail.
I have a suggestion regarding the discussion section of the manuscript. Furthermore, I believe my advice could be valuable to the Scottish lung cancer screening program from a technical standpoint and help increase the response rate among invited individuals.
The cornerstone of any program's success is high participation among invited individuals.
Results from other studies show that when people are invited by phone or during an in-person visit, participation rates are much higher.

https://doi.org/10.1016/j.ctarc.2025.100889

https://doi.org/10.3390/cancers17121956

Thank you for your positive feedback and helpful insights. We are grateful for this helpful suggestion. We agree that trusted endorsement is likely to be an important factor in engaging marginalised groups and have expanded our discussion on page 14 accordingly. We have also included one of your suggested references to strengthen this section. We have also included one of your suggested references to provide further recent evidence on this point and strengthen this section.

We also have some qualitative findings that we are reporting in a separate manuscript that touch on the role of trust in health services as an important facilitator to engagement with them (including participation in screening).

Reviewer: 3
Dr. Geertruida H. de Bock, University Medical Center Groningen
Comments to the Author:
Review
This study tested if risk based lung cancer screening using low dose CT could be delivered across different regions in Scotland. The programme worked ok for people who took part actually, showing high rates of lung cancer detection and also many previously unknown lung and heart conditions, but overall participation was low (about 24%), and especially in more deprived areas and in higher risk groups like current smokers uptake was poor.

I have some problems with the conclusion that the programme is feasible. It is mainly useful for the people who comes to take part, but not really for the whole population, because the highest risk people are less likely to take part. Maybe it should be more clearly explained what “feasible” means in this context. Is it only that the programme can be organised and run in practice?
Thank you very much for your feedback on our manuscript and your comments to improve it. We are grateful for this thoughtful comment. We agree that the distinction between feasibility of programme delivery and effectiveness as a population screening programme needed to be clearer. We have therefore revised both the discussion and conclusions (pages 12 and 15) to make this distinction more explicit and to moderate our conclusions accordingly i.e. the practical steps of identifying, inviting and screening individuals at high risk, and toning down the reach of our conclusions. We have also addressed your concerns in these sections in relation to your point below.

My feeling is that this study shows LDCT screening can be delivered in practice and can find important disease in a high risk subset, but it does not yet show that it works well as a population screening programme in a fair and effective way. The main problem is not the scanning itself, it is more getting the people who are most at risk to actually join, and also how to deal with all the follow up from so many abnormal results.
Thank you. You have raised important challenges for the delivery of lung cancer screening. We hope our revisions throughout the manuscript now more accurately reflect the scope of our feasibility

study and the outstanding issues facing implementation of lung screening in Scotland and beyond. Large scale implementation has begun in England, where uptake rates are approaching 50%. We would recommend that we start a programme to mirror this in Scotland and then iteratively target at risk populations to further increase uptake in communities with the greatest need.

Even for people who did join, it can be asked if this is always a good screening test. The yield is high for lung cancer and other lung findings, so targeting seems good. But there is also a very high number of "abnormal findings", which can mean overdiagnosis burden, more follow up tests, anxiety for patients, and higher healthcare costs. Also for some of these findings we are not really knowing what the best treatment is.

We would fully agree that these are also important implementation challenges for a lung cancer screening programme and appreciate you highlighting them. We have revised the discussion to place greater emphasis on these issues and to acknowledge that they represent important considerations for future implementation of lung cancer screening programmes.

I would also like to ask the authors to maybe rethink the strengths and limitations section after the abstract. Maybe I missed it, but what was done when the different risk tools did not agree? In table 2 many patients did not meet the criteria. What are the consequences of this? Was there less cancer in the group where criteria were not met?

Thank you for highlighting this. Participants were offered a LDCT scan if they met the criteria for any of the three risk tools, so that the lowest threshold was implemented. This is stated on page 5 under 'risk assessment' but we have edited this slightly to emphasise this point. We don't have the cancer outcomes for those who did not meet the criteria.

What was the follow-up time? Or was this only the findings on the first round? It could be the case that the lung cancer detection rate will be higher, as the diagnostic testing is not finished in all patients yet.

Yes, we were only able to offer one round of screening as part of our study, and the follow-up ended with the end of the study. We have acknowledged this in the study limitations.

VERSION 2 - REVIEW

**Reviewer**	**1**
**Name**	**Philip, Keir**
**Affiliation**	**National Heart and Lung Institute**
**Date**	**04-Aug-2026**
**COI**	

Many thanks for addressing points raised.

**Reviewer**	**3**
**Name**	**de Bock, Geertruida H.**

**Affiliation**	**University Medical Center Groningen**
**Date**	**06-Aug-2026**
**COI**	

---

well done
