# Supplementary material for: Pilot feasibility study to deliver low-dose CT lung cancer screening in Scotland: descriptive findings from the LungScot study
Source: BMJ Open. 2026 Sep 15;16(9):e120569. doi: 10.1136/bmjopen-2026-120569 (PMC13583738; doi:10.1136/bmjopen-2026-120569)
Supplement: Supplementary file 2 [file bmjopen-16-9-s001.docx]

|  | **Practice 1** | **Practice 2** | **Practice/Mailout 3** | **Practice 4** | **Practice 5** | **Practice 6** | **Practice 7** | **Totals** | **Median age (years)** |
| --- | --- | --- | --- | --- | --- | --- | --- | --- | --- |
| **Health Boards** |  |  |  |  |  |  |  | 2801 invited, 668 responses **23.8%** | 63y |
| **Lothian** | 259 invited, 64 responses  **24.7%** | 218 invited, 83 responses  **38.1%** | 249 invited, 46 responses  **18.5%** | 198 invited, 45 responses  **22.7%** | 239 invited, 44 responses  **18.4%** | 117 invited, 33 responses  **28.2%** | 248 invited, 64 responses  **25.8%** | 1528 invited, 379 responses  **24.8%** | 63y |
| **Lothian SIMD quintiles** | 1 – 0, 0 0% 2 – 13, 21%  3 – 20, 32.3%  4- 18, 29%  5- 11, 17.7% | 1- 9, 11.1% 2- 25, 30.9% 3- 13, 16% 4- 23, 28.4% 5- 11, 13.6% | 1- 15, 32.6% 2- 25, 54.3% 3- 5, 8.3% 4- 1, 2.2% 5- 0, 0% | 1- 1, 2.7% 2- 16, 36.4% 3- 17, 38.6% 4- 10, 22.7% 5-0, 0% | 1- 2, 4.9% 2- 12, 29.3% 3- 14, 34.1% 4- 8, 19.5% 5- 5, 12.2% | 1- 0, 0% 2- 3, 9.1% 3- 8, 24.2% 4- 8, 24.2% 5- 14, 42.4% | 1- 0, 0% 2- 16, 25.4% 3- 26, 41.3% 4- 17, 27% 5- 4, 6.3% | 1- 27, 7.3% 2- 109, 29.5 % 3- 103, 27.8% 4- 84, 21.9% 5- 45, 13.2% (n=11 missing data) |  |
| **Lothian  no. existing health conditions** | 0- 29, 45.3% 1- 21, 32.8% 2- 7, 10.9% 3- 4, 6.3% 4- 3, 4.7% 5+ - 0, 0% | 0- 40, 48.2% 1- 25, 30.1% 2- 10, 12% 3- 7, 8.4% 4- 0, 0% 5+ - 1, 1.2% | 0- 11, 23.9% 1- 15, 32.6% 2- 15, 32.6% 3- 3, 6.5% 4- 2, 4.3% 5+ - 0, 0% | 0- 19. 42% 1- 16, 35.6% 2- 5, 11.1% 3- 3, 6.7% 4- 2, 4.4% 5+ - 0, 0% | 0- 21, 47.7% 1- 13, 29.5% 2- 9, 20.5% 3- 1, 2.3% 4- 0, 0% 5+- 0, 0% | 0- 18, 54.5% 1- 7, 21.1% 2- 6, 18.1% 3- 2, 6.1% 4- 0, 0% 5+ - 0, 0% | 0- 31, 48.4% 1- 18, 28.1% 2- 8, 12.5% 3- 6, 9.4% 4- 1, 1.6% 5+ - 0, 0% | 0- 169, 44.6% 1- 115, 30.4% 2- 60, 15.8% 3- 26, 6.9% 4- 8, 2.1% 5+ - 1, 0.3% |  |
|  |  |  |  |  |  |  |  |  |  |
| **Greater Glasgow and Clyde (GGC)** | 235 invited, 44 responses  **18.7%** | 183 invited, 33 responses  **18%** |  |  |  |  |  | 418 invited, 77 responses  **18.35%** | 62y |
| **GGC SIMD quintiles** | 1- 34, 77.3% 2- 7, 15.9% 3- 2, 4.5% 4- 0, 0% 5- 1, 2.3% | 1- 19, 59.4% 2- 9, 28.1% 3- 1, 3.1% 4- 2, 6.3% 5- 1, 3.1% |  |  |  |  |  | 1- 53, 68.4% 2- 16, 22.0% 3- 3, 3.9% 4- 2, 2.6% 5- 2, 2.6% (n=1 missing) |  |
| **GGC no. existing health conditions** | Data not available | Data not available |  |  |  |  |  |  |  |
|  |  |  |  |  |  |  |  |  |  |
| **Grampian** | 101 invited, 18 responses  **17.8%** | 112 invited, 20 responses  **17.9%** | 100 invited, 21 responses  **21%** |  |  |  |  | 313 invited, 59 responses  **18.9%** | 63y |
| **Grampian SIMD quintiles** | 1- 13, 72.2% 2- 3, 16.7% 3- 1, 5.6% 4- 1, 0% 5- 0, 5.6% | 1- 5, 25% 2- 7, 35% 3- 4, 20% 4- 4, 20% 5- 0, 0% | 1- 0, 0% 2- 2, 9.5% 3- 16, 76.2% 4- 3, 14.3% 5- 0, 0% |  |  |  |  | 1- 18, 30.5% 2- 12, 20.3% 3- 21, 35.6% 4- 8, 13.6% 5- 0, 0% |  |
| **Grampian no. existing health conditions** | 0- 9, 50% 1- 4, 22.2% 2- 1, 5.6% 3- 2, 11.1% 4- 1, 5.6% 5+ - 1, 5.6% | 0- 4, 20% 1- 8, 40% 2- 5, 25% 3- 2, 10% 4- 1, 5% 5+ - 0, 0% | 0- 4, 19% 1- 10, 47.6% 2- 4, 19% 3- 2, 9.5% 4- 0, 0% 5+ - 1, 4.8% |  |  |  |  | 0- 17, 28.8% 1- 22, 37.3% 2- 10, 16.9% 3- 6, 10.2% 4- 2, 3.4% 5+ - 2, 3.4% |  |
|  |  |  | Practice 2 (2^nd^ mailout) |  |  |  |  |  |  |
| **Highlands** | 97 invited, 34 responses  **35.1%** | 250 invited, 52 responses  **20.8%** | 195 invited, 67 responses  **34.3%** |  |  |  |  | 542 invited, 153 responses  **30.1%** | 62y |
| **Highlands SIMD quintiles** | 1- 0, 0% 2- 0, 0% 3- 18, 52.9% 4- 16, 47.1% 5- 0, 0% | 1- 1, 1.9% 2- 25, 48.1% 3- 16, 30.8% 4- 10, 19.2% 5- 0, 0% | 1- 13, 19.4% 2- 20, 29.9% 3- 17, 25.4% 4- 17, 25.4% 5- 0, 0% |  |  |  |  | 1- 14, 9.2% 2- 45, 29.4% 3- 51, 33.3% 4- 43, 28.1% 5- 0, 0% |  |
| **Highlands no. existing health conditions** | 0- 16, 47.1% 1- 8, 23.5% 2- 7, 20.6% 3- 2, 5.9% 4- 1, 2.9% 5+ - 0, 0% | 0- 29, 55.8% 1- 13, 25% 2- 5, 9.6% 3- 4, 7.7% 4- 0, 0% 5+ - 1, 1.9% | 0- 37, 55.2% 1- 18, 26.9% 2- 9, 13.4% 3- 1, 1.5% 4- 2, 3% 5+ - 0, 0% |  |  |  |  | 0- 82, 53.4% 1- 39, 25.5% 2- 21, 13.7% 3- 7, 4.6% 4- 3, 2.0% 5+ - 1, 0.7% |  |
