# Supplementary material for: Pilot feasibility study to deliver low-dose CT lung cancer screening in Scotland: descriptive findings from the LungScot study
Source: BMJ Open. 2026 Sep 15;16(9):e120569. doi: 10.1136/bmjopen-2026-120569 (PMC13583738; doi:10.1136/bmjopen-2026-120569)
Supplement: Supplementary file 3 [file bmjopen-16-9-s002.docx]

**Supplementary material table 2: Self-reported smoking among participants by health board region**

| **Health Board** |  | **Past Smokers** | **Current smokers** |
| --- | --- | --- | --- |
| **Lothian** |  | 185 (54.4%) | 155 (45.6%) |
| **GGC** |  | 38 (54.3%) | 32 (45.7%) |
| **Grampian** |  | 19 (33.3%) | 38 (66.7%) |
| **Highlands** |  | 63 (44.7%) | 78 (55.3%) |
| **Totals** |  | **305 (50.2%)** | **303 (49.8%)** |
