# Supplementary material for: Pilot feasibility study to deliver low-dose CT lung cancer screening in Scotland: descriptive findings from the LungScot study
Source: BMJ Open. 2026 Sep 15;16(9):e120569. doi: 10.1136/bmjopen-2026-120569 (PMC13583738; doi:10.1136/bmjopen-2026-120569)
Supplement: Supplementary file 4 [file bmjopen-16-9-s003.docx]

**Supplementary Table 3: Breakdown of risk assessment by tool for high-risk participants (n=503)**

|  | **Threshold met by one tool only** | **Threshold met by two tools only** | | **Threshold met by all three tools** | **Totals** |
| --- | --- | --- | --- | --- | --- |
| **Risk tool** |  |  | **Combinations** |  |  |
| **LLPv2** | 45 (8.9%) | 48 | 15 w/ PLCO | 282 (56.1%) | 375 (74.6%) |
|  |  |  | 33 w/ USPSTF |  |  |
| **PLCOm2012** | 3 (0.6%) | 77 | 15 w/ LLP | 282 | 362 (72.0%) |
|  |  |  | 62 w/ USPSTF |  |  |
| **USPSTF** | 63 (12.5%) | 95 | 33 w/ LLP | 282 | 440 (87.5%) |
|  |  |  | 62 w/ PLCO |  |  |
