# Supplementary material for: Pilot feasibility study to deliver low-dose CT lung cancer screening in Scotland: descriptive findings from the LungScot study
Source: BMJ Open. 2026 Sep 15;16(9):e120569. doi: 10.1136/bmjopen-2026-120569 (PMC13583738; doi:10.1136/bmjopen-2026-120569)
Supplement: Supplementary file 5 [file bmjopen-16-9-s004.docx]

**Supplementary Table 4: Comparing responder and non-responder characteristics: Sex and age**

| **Health Board** |  | **Responder males** | **Non-responder**  **Males** | **Responder females** | **Non-responder**  **Females** | **Responders average age** | **Non-responder average age (years)** |
| --- | --- | --- | --- | --- | --- | --- | --- |
| **Lothian** |  | 171 (50.3%) | 593  (51.5%) | 169 (49.7%) | 558  (48.5%) | 63y | 63y |
| **GGC** |  | 30  (42.9%) | 168  (49.3%) | 40  (57.1%) | 173  (50.7%) | 62y | 63y |
| **Grampian** |  | 21  (36.8%) | 132  (52.0%) | 36  (63.2%) | 122  (48.0%) | 63y | 62y |
| **Highlands** |  | 77  (54.6%) | Data not available | 64  (45.4%) | Data not available | 62y | 63y |
| **Totals** |  | **299 (49.2%)** | **893 (51.1%)** | **309 (50.8%)** | **853 (48.9%)** | **63y** | **63y** |

**Supplementary Table 5: Comparing responder and non-responder characteristics: Smoking behaviour**

| **Health Board** |  | **Responder ever smokers** | **Non-responder**  **Ever Smokers** | **Responder current smokers** | **Non-responder**  **Current smokers** |
| --- | --- | --- | --- | --- | --- |
| **Lothian** |  | 185 (54.4%) | 387 (33.7%) | 155 (45.6%) | 763 (66.3%) |
| **GGC** |  | 38 (54.3%) | Data not available | 32 (45.7%) | Data not available |
| **Grampian** |  | 19 (33.3%) | 58 (22.8%) | 38 (66.7%) | 196 (77.2%) |
| **Highlands** |  | 63 (44.7%) | 106 (27.2%) | 78 (55.3%) | 283 (72.8%) |
| **Totals** |  | **305 (50.2%)** | **551 (30.7%)** | **303 (49.8%)** | **1242 (69.3%)** |

**Supplementary Table 6: Comparing responder and non-responder characteristics: SIMD quintiles (Non-responders: n=2121, missing data n=10; Responders: n=655, missing data n=12)**

| **Health Board** |  | **1** | **2** | **3** | **4** | **5** |
| --- | --- | --- | --- | --- | --- | --- |
| **Lothian** | Responders | 27 (7.3%) | 109 (29.5%) | 103 (27.8%) | 84  (21.9%) | 45  (13.2%) |
|  | Non-responders | 107  (9.4%) | 361  (31.6%) | 396 (34.7%) | 197  (17.3%) | 80  (7.0%) |
| **GGC** | Responders | 53 (68.4%) | 16 (22.0%) | 3 (3.8%) | 2 (2.6%) | 2 (2.6%) |
|  | Non-responders | 243  (72.1%) | 59  (17.5%) | 11  (3.3%) | 16  (4.7%) | 8  (2.4%) |
| **Grampian** | Responders | 18 (30.5%) | 12 (20.3%) | 21 (35.6%) | 7 (13.6%) | 0 (0%) |
|  | Non-responders | 80  (31.5%) | 83  (32.7%) | 57  (22.4%) | 20  (7.9%) | 14  (5.5%) |
| **Highlands** | Responders | 14 (9.2%) | 45 (29.4%) | 51 (33.3%) | 43 (28.1%) | 0 (0%) |
|  | Non-responders | 41  (10.5%) | 107  (27.5%) | 145  (37.3%) | 96  (24.7%) | 0  (0%) |
| **Totals** | Responders | 112 (17.1%) | 182 (27.8%) | 178 (27.2%) | 136 (20.8%) | 47 (7.2%) |
|  | Non-responders | 471 (22.2%) | 610  (28.8%) | 609  (28.7%) | 329  (15.5%) | 102  (4.8%) |

**Supplementary Table 7: Comparing responder and non-responder characteristics: Existing health conditions (n=1756, missing data n=375; Responders n=591, missing data=77)**

| **Health Board** |  | **0** | **1** | **2** | **3** | **4** | **5+** |
| --- | --- | --- | --- | --- | --- | --- | --- |
| **Lothian** | Responders | 169 (44.6%) | 115 (30.3%) | 60 (15.8%) | 26  (6.9%) | 8  (2.1%) | 1 (0.3%) |
|  | Non-responders | 566  (49.2%) | 297  (25.8%) | 174  (15.1%) | 69  (6.0%) | 29  (2.5%) | 15  (1.3%) |
| **GGC** | Responders | Data not available | Data not available | Data not available | Data not available | Data not available | Data not available |
|  | Non-responders | Data not available | Data not available | Data not available | Data not available | Data not available | Data not available |
| **Grampian** | Responders | 17 (28.8%) | 22 (37.3%) | 10 (16.9%) | 6 (10.2%) | 2 (3.4%) | 2 (3.4%) |
|  | Non-responders | 130  (51.2%) | 63  (24.8%) | 35  (13.8%) | 16  (6.3%) | 8  (3.1%) | 2  (0.8%) |
| **Highlands** | Responders | 82 (53.4%) | 39 (25.5%) | 21 (13.7%) | 7 (4.6%) | 3 (2.0%) | 1 (0.7%) |
|  | Non-responders | 164  (42.2%) | 107  (27.5%) | 49  (12.6%) | 24  (6.2%) | 5  (1.3%) | 3  (0.8%) |
| **Totals** | Responders | **268 (45.3%)** | **176 (29.8%)** | **91 (15.4%)** | **39 (6.6%)** | **13 (2.2%)** | **4 (0.7%)** |
|  | Non-responders | **860**  **49.0%** | **467**  **26.6%** | **258**  **14.7%** | **109**  **6.2%** | **42**  **2.4%** | **20**  **1.1%** |
